# Evolution of the leukotoxin promoter in genus *Mannheimia*

**DOI:** 10.1186/1471-2148-9-121

**Published:** 2009-05-29

**Authors:** Jesper Larsen, Anders G Pedersen, Robert L Davies, Peter Kuhnert, Joachim Frey, Henrik Christensen, Magne Bisgaard, John E Olsen

**Affiliations:** 1 Department of Veterinary Pathobiology, Faculty of Life Sciences, University of Copenhagen, Stigbøjlen 4, DK-1870 Frederiksberg C, Denmark; 2 Center for Biological Sequence Analysis, BioCentrum-DTU, Technical University of Denmark, Building 208, DK-2800 Lyngby, Denmark; 3 Institute of Biomedical and Life Sciences, Glasgow Biomedical Research Centre, University of Glasgow, 120 University Place, Glasgow G12 8TA, UK; 4 Institute of Veterinary Bacteriology, University of Berne, Länggass-Strasse 122, CH-3012 Berne, Switzerland

## Abstract

**Background:**

The *Mannheimia *species encompass a wide variety of bacterial lifestyles, including opportunistic pathogens and commensals of the ruminant respiratory tract, commensals of the ovine rumen, and pathogens of the ruminant integument. Here we present a scenario for the evolution of the leukotoxin promoter among representatives of the five species within genus *Mannheimia*. We also consider how the evolution of the leukotoxin operon fits with the evolution and maintenance of virulence.

**Results:**

The alignment of the intergenic regions upstream of the leukotoxin genes showed significant sequence and positional conservation over a 225-bp stretch immediately proximal to the transcriptional start site of the *lktC *gene among all *Mannheimia *strains. However, in the course of the *Mannheimia *genome evolution, the acquisition of individual noncoding regions upstream of the conserved promoter region has occurred. The rate of evolution estimated branch by branch suggests that the conserved promoter may be affected to different extents by the types of natural selection that potentially operate in regulatory regions. Tandem repeats upstream of the core promoter were confined to *M. haemolytica *with a strong association between the sequence of the repeat units, the number of repeat units per promoter, and the phylogenetic history of this species.

**Conclusion:**

The mode of evolution of the intergenic regions upstream of the leukotoxin genes appears to be highly dependent on the lifestyle of the bacterium. Transition from avirulence to virulence has occurred at least once in *M. haemolytica *with some evolutionary success of bovine serotype A1/A6 strains. Our analysis suggests that changes in *cis*-regulatory systems have contributed to the derived virulence phenotype by allowing phase-variable expression of the leukotoxin protein. We propose models for how phase shifting and the associated virulence could facilitate transmission to the nasopharynx of new hosts.

## Background

The genus *Mannheimia *includes strains previously classified as trehalose-negative [*Pasteurella*] *haemolytica *and is one of the most well-defined and robust clusters within the gamma-proteobacterial family of *Pasteurellaceae *Pohl 1981 [1]. The *Mannheimia *species have taken divergent paths toward their distinct lifestyles. The majority of strains isolated from pulmonary infection in cattle belongs to *M. haemolytica *serotype A1/A6 [2]. These strains are sub-dominant to other serotypes (e.g., bovine serotype A2 strains) in the nasopharynx of healthy cattle but dominate when the host defences are at least partly compromised [3-5]. In the immunocompromised host, they have an increased capacity for proliferation and can achieve relatively high total numbers in the nasopharynx, where they are likely to be transmitted to the nasopharynx of new hosts or to spill over or otherwise enter the lungs [6]. However, pulmonary infection caused by *M. haemolytica *serotype A1 is considered to be non-communicable (i.e. no direct transmission between the lungs) and the continuous circulation of these bacteria in bovine populations seems to depend on their capacity for asymptomatic transmission to the nasopharynx, and not the lungs, of new hosts [7]. By contrast, bovine serotype A2 strains of *M. haemolytica *have had a long history in bovine populations, where they colonise the nasopharynx asymptomatically and rarely cause disease [2]. Also strains of *M. glucosida *serotype A11 and *M. ruminalis*, the sister group to *M. haemolytica *+ *M. glucosida*, appear to be adapted to a relatively benign lifestyle in the ovine nasopharynx and rumen, respectively [1,2]. In addition, bovine strains of *M. varigena *biogroup 6, which is the most basal of the *Mannheimia *species, colonise the nasopharynx asymptomatically, although they have the propensity to cause disease [1]. It therefore seems reasonable to conclude that the genes responsible for the virulence of *Mannheimia *strains must evolve in response to the demands (selection pressures) associated with a commensal lifestyle rather than any advantages that might arise from causing disease.

Previous works have revealed that the leukotoxin (LktA) protein produced by *M. haemolytica *serotype A1 provides protection from the circulating constitutive and inducible immune defences during pulmonary infection via interactions with host cells [8-10]. Interestingly, much of the virulence can be blamed on the seemingly misguided overresponse of the immune defences [11]. The LktA protein induces the overwhelming activation of neutrophils leading to production of cytokines that mediate tissue injury [12-15].

The leukotoxin (*lkt*) operon codes for four proteins: an internal acyltransferase, encoded by *lktC *[16]; the structural toxin, encoded by *lktA*, which belongs to the *Escherichia coli *HlyA-like subfamily of cytotoxic RTX (repeats in toxin) proteins [16]; an inner membrane protein with a cytoplasmic ATP-binding cassette (ABC) domain, encoded by *lktB*, which pumps out the LktA protein via interaction with the C terminus of the LktA protein [17]; and a membrane fusion protein, encoded by *lktD*, which forms a bridge between the inner and outer membranes [17]. The genes for these four proteins are physically adjacent on the chromosome and are transcribed as *lktCA *or *lktCABD *messages [18,19].

Recent reports provided the first support for the view that the *lkt *operon was vertically inherited from the last common ancestor of genus *Mannheimia *to any ancestor of the diverging species, while changes such as chromosomal rearrangements, pseudogene formation, and deletions of various sizes have occurred in the region upstream of the *lkt *genes in individual subclades/species [20,21]. However, the extent to which these molecular evolutionary forces affect *lkt *gene regulation is not known.

In this article, we analysed the *lkt *promoter among representatives of the five *Mannheimia *species in order to identify changes in *cis*-regulatory systems that could have catalysed adaptive evolution. We started our study using multiple sequence alignment as a tool for the identification of orthologous sequences in the intergenic region upstream of the *lkt *genes (called hereafter the conserved promoter, CP). We also surveyed existing data on regulatory sequences in order to delineate the spatial and functional boundaries of the CP region. On the basis of the multiple sequence alignment, we estimated the rate of evolution for each CP sequence in order to infer the selective constraints. We finally consider how the evolution of the *lkt *operon fits with the evolution and maintenance of virulence.

## Results and discussion

Table 1 presents the *Mannheimia *strains analysed in this study, sorted by their overall similarity to *M. haemolytica *serotype A1 str. PHL213. This measure of similarity is based on 16S rRNA phylogenies [1,20] and on relationships resolved by multilocus enzyme electrophoresis (MLEE) typing within *M. haemolytica *+ *M. glucosida *[2].

**Table 1:** Strains used in this study

Subclade^a^	Taxon^b^	Strain ID	Serotype	Host	Country	GenBank accession no.^c^	
						
						16S rRNA	*lkt *promoter
I	Biogroup 1	PHL213	A1	*Bos taurus*			
I	Biogroup 1	CCUG 12392^T^	A2	*Ovis aries*	UK	[GenBank:AF060699]	[GenBank:AY425276]
							
	*M. glucosida*						
I	Biogroup 3B	P925^T^	A11	*Ovis aries*	Scotland	[GenBank:AF053889]	[GenBank:AY425277]
							
	*M. ruminalis*						
II	Bt 18 biovar 2	HPA113		*Ovis aries*	UK	[GenBank:AY425283	[GenBank:AY425280]
							
	*M. granulomatis*						
III	[*P*.] *granulomatis*	P1135/26^T^		*Bos taurus*	Brazil	[GenBank:AF053902]	[GenBank:AY425278]
							
	*M. varigena*						
IV	Biogroup 6	177^T^		*Bos taurus*	Germany	[GenBank:AF053893]	[GenBank:AY425279]

^a ^Subclades refer to monophyletic groups based on phylogenetic analysis of 16S rRNA sequences [1,20].

^b ^Bt = Bisgaard taxon.

^c ^These sequences have been published elsewhere.

### Conservation and flexibility in the intergenic region upstream of the *lkt *genes

**Figure 1:**
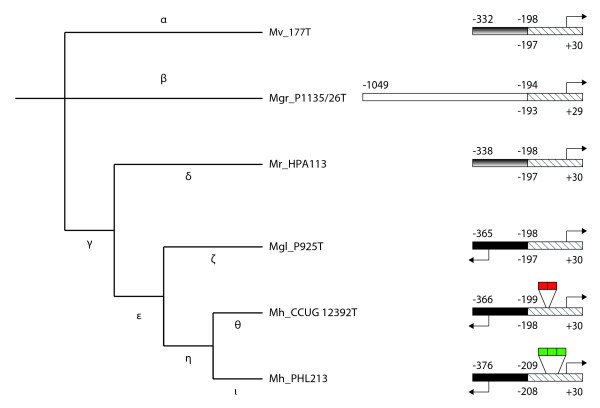
**Structure and evolution of the intergenic regions upstream of the *lkt *genes**. Cladogram that describes bifurcation order is based on 16S rRNA phylogenies and MLEE. Greek letters indicate individual branches. The CP region (sequence shared among all *Mannheimia *strains) is hatched. The site of the repeat sequences in the CP region is indicated by an interruption in hatched box and the number of imperfect and perfect repeat units per promoter is indicated by red and green blocks, respectively. The ancient *lapB *region is presented by fountain filled box. Individual noncoding regions upstream of the CP region in *M. haemolytica *+ *M. glucosida *and *M. granulomatis *are shown by black and white boxes, respectively. Arrows denote the putative transcription start sites. Mh, *M. haemolytica*: Mgl; *M. glucosida*; Mr, *M. ruminalis*; Mgr, *M. granulomatis*; Mv, *M. varigena*.

**Figure 2:**
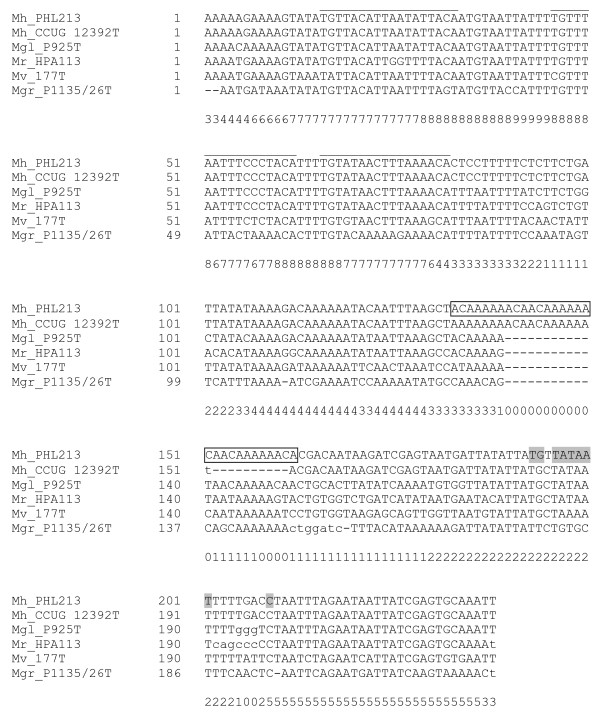
**Alignment of the CP region**. The alignment of the intergenic regions upstream of the *lkt *genes showed significant sequence and positional conservation over a 225-bp stretch immediately proximal to the transcriptional start site of the *lktC *gene among all *Mannheimia *strains (CP region), with the notable exception of the region corresponding to the repeat tract in *M. haemolytica *serotype A1 [22], which accommodated many sequence variations. Capital letters denote aligned residues. Lower-case letters are not considered to be aligned. Numbers (0–9) below the alignment reflect the relative degree of local similarity among the sequences. Previous works [18,22,23] allowed us to infer *cis*-regulatory sequences. The footprinted palindromic motifs are indicated by an over line. The repeat sequences are enclosed by a rectangle. The core promoter including the -10 hexamer, the extended TG motifs, and the transcription start site (+1) is highlighted. Mh, *M. haemolytica*: Mgl; *M. glucosida*; Mr, *M. ruminalis*; Mgr, *M. granulomatis*; Mv, *M. varigena*.

The alignment revealed 100% identity over the entire 406-bp intergenic region from *M. haemolytica *serotype A1 str. PHL101 and str. PHL213 (data not shown) and previous reports [18,22,23] allowed us to infer *cis*-regulatory sequences. The 406-bp region was shared among *M. haemolytica *serotype A2 and *M. glucosida *serotype A11 as reflected in Figure 1. The alignment showed significant sequence and positional conservation over a 225-bp stretch immediately proximal to the transcriptional start site of the *lktC *gene among all *Mannheimia *strains (CP region), with the notable exception of the region corresponding to the repeat tract in *M. haemolytica *serotype A1 [22], which accommodated many sequence variations (Figures 1 and 2).

In *M. haemolytica *+ *M. glucosida*, the distal 180-bp stretch of sequence was juxtaposed to the transcriptional start site of the *artJ *gene (Figure 1). No homology was detected between this 180-bp stretch and the noncoding regions upstream of the CP region in the remaining *Mannheimia *species (data not shown).

In order to gain some insight into the possible evolutionary mechanisms of this differential pattern, we performed for each species analysis of sequence and positional conservation in the noncoding region upstream of the CP region. In *M. ruminalis *and *M. varigena*, these regions contained stretches of sequence unique to the *lapB *genes (data not shown). The region of *M. granulomatis *was adjacent to the *xylB *pseudogene and contained a fragment of about 620 bp corresponding to the distal region of the parental *xylB *gene as described elsewhere [21]. Moreover, this region contained a 50-bp stretch of inverted sequence that was homologous to the *hslV*-*hslU *intergenic regions and stretches of sequence unique to the *lapB *genes (data not shown).

These observations provide support for the view that the *artJ *operon, including 180-bp of sequence upstream of the transcribed *artJ *gene, was translocated into the intergenic region upstream of the *lkt *genes during early evolution of *M. haemolytica *+ *M. glucosida *and that the *hslVU *and *lapB *operons has undergone pseudogene formation and deletions in *M. granulomatis *after divergence from the remaining *Mannheimia *species [21].

These observations illustrate how a few evolutionary events in closely related strains can alter dramatically an intergenic region. In the case of *M. haemolytica *+ *M. glucosida*, the alteration coincided with an evolutionary success. Interestingly, transcriptional coupling has been reported for the divergently transcribed *artJ *and *lkt *promoters [23]. However, the role of this regulatory feature during transmission of bacteria through host populations is not known. The chimeric promoter was found in all *M. haemolytica *+ *M. glucosida *strains in the present study, irregardless of their propensity to cause disease, suggesting that it evolved in response to selection for something else than making their hosts sick.

### Evolutionary rates for CP sequences

The role for natural selection in the evolution of regulatory regions is quite unclear [24]. Recognisable features and motifs are often labile, small in size, and may depend on the sequence context [25,26]. Therefore, the complex structure of these regions could be due to alternative evolutionary forces [27-30]: (i) compensatory selection occurring when a pair of mutations at different sites that would be singly deleterious produces normal fitness in combination; (ii) stabilising selection could maintain stable levels of gene expression while allowing mutational turnover of functional important sites; (iii) purifying selection could facilitate maintenance of specific functional sites.

The evolutionary rates for CP sequences were tested for deviations from expectations derived from the null hypothesis of neutral evolution. The overall length of the 16S rRNA tree (obs. *T*_16S_) was 0.102024 substitutions per site, while the overall length of the CP tree (obs. *T*_CP_) was 1.33986 substitutions per site. The null hypothesis predicts that the expected length of any given branch on the CP tree (exp. *v*_CP_) can be calculated by multiplying the observed length of the corresponding branch on the 16S rRNA tree (obs. *v*_16S_) by 1.33986/0.102024 = 13.13292. The observed and the expected lengths for any given branch on the CP tree and their pairwise differences are summarised in Table 2.

**Table 2:** Evolutionary rate differences for CP sequences

Branch^a^	Branch lengths				*p *value^b^	Significance^c^
			
	Obs. *ν*_16S_	Obs. *ν*_CP_	Exp. *ν*_CP_	Obs. *ν*_CP _– Exp. *ν*_CP_		
α	0.018827	0.247291	0.247253	0.000038	0.992400	
β	0.023416	0.720649	0.307520	0.413129	0.002200	Obs. *ν*_CP _> Exp. *ν*_CP_
γ	0.015113	0.000000	0.198478	-0.198478	<0.000100	Obs. *ν*_CP _< Exp. *ν*_CP_
δ	0.006960	0.117649	0.091405	0.026244	0.490000	
ε	0.008202	0.074547	0.107716	-0.033169	0.434800	
ζ	0.003357	0.103029	0.044087	0.058942	0.046800	
η	0.015106	0.062805	0.198386	-0.135581	0.000400	Obs. *ν*_CP _< Exp. *ν*_CP_
θ	0.002232	0.005772	0.029313	-0.023541	0.269600	
ι	0.008811	0.008115	0.115714	-0.107599	<0.000100	Obs. *ν*_CP _< Exp. *ν*_CP_

^a ^Greek letters indicate individual branches as in Figure 1.

^b ^Two-tailed *p*-value.

^c ^Two-tailed *p*-values < 0.0056 adjusted by the Bonferroni correction for multiple tests were considered significant.

For three of four branches ancestral to *M. haemolytica *serotype A1, the differences between the observed and the expected branch lengths were significantly smaller than zero (the value expected under the null hypothesis of neutral evolution) (Table 2). This is in accord with the operation of stronger levels of purifying selection and possibly reflects an ancient balance between the immune system and these primarily commensal bacteria and their expression of the LktA protein during asymptomatic colonisation of the nasopharynx. The difference between the observed and the expected branch lengths ancestral to *M. haemolytica *+ *M. glucosida *was not significantly different from zero (Table 2). This coincided with the translocation of the *artJ *operon, including 180-bp of sequence upstream of the transcribed *artJ *gene, into the intergenic region upstream of the *lkt *genes [21]. It is possible that this relatively short window of time was dominated by stabilizing selection on this chimeric region, maintaining stable levels of *lkt *(and *artJ*) expression and thus the ancient balance between the immune system and bacteria during asymptomatic colonisation of the nasopharynx.

The difference between the observed and the expected branch lengths ancestral to *M. granulomatis *was significantly greater than zero (Table 2). Moreover, the CP region appeared to be more variable in *M. granulomatis*, especially in terms of the overall conservation of *cis*-regulatory sequences (Figure 2). Most notably, there was a high level of sequence degeneracy in the core promoter, which was highly conserved among the other *Mannheimia *species. Strains belonging to the bovine taxon of *M. granulomatis*, including *M. granulomatis *str. P1135/26^T^, cause severe skin infection (lechiguana) [1,31,32]. Interestingly, these strains have never been isolated from healthy cattle, including the nasopharynx, and the disease seems to be acquired by contact with the human botfly (*Dermatobia hominis*), i.e. there is no direct transmission between cattle or transmission is short-lived [33]. Moreover, comparison of *lktA *genes from different taxa within *M. granulomatis *have shown that those from bovine strains are under significantly relaxed selective constraints (J. Larsen, unpublished results). We propose that genetic diversity of the CP sequence in this strain reflects functional decay due to relaxed selection outside the nasopharynx, enabling mutations to accumulate in the portions of the genome that encode unused functions in the integumentary system. However, it is also possible that positive Darwinian selection for alternative *cis*-regulatory sequences has acted as a mechanism for coordinating expression of the LktA protein with the nutritional and environmental conditions of the integument. For example, Larsen et al. [20] have shown that the β-haemolytic phenotype, a marker of the *lktA *genotype in *M. haemolytica *[34], is present in this strain, suggesting at least some functional conservation of the *lkt *operon.

### Evolution of repeat sequences

Tandem repeats of 5'-ACAAAAAACA-3' upstream of the core promoter were first identified in *M. haemolytica *serotype A1 str. PHL101 [22]. Our preliminary analysis revealed three and two repeat units per promoter in *M. haemolytica *serotype A1 and serotype A2, respectively (Figure 1). In the latter case, the two repeat units varied from the consensus sequence by one nucleotide change each (Figure 2). Because this region accommodated many sequence variations in the remaining *Mannheimia *species, the unit sequence could not be determined, suggesting that repeat sequences are confined to *M. haemolytica*.

The relatively recent appearance of three repeat units (at least after the divergence of *M. haemolytica *serotype A1 and serotype A2 as reflected in Figure 1) may provide us with an opportunity to trace the entire evolutionary process back to its origin. Therefore, we examined the number of repeat units per promoter in 28 additional *M. haemolytica *strains, which represent the diversity within this species based on MLEE, geographic origin, and host association (Table 3). The distribution of the number of repeat units per promoter showed that there were three or more repeat units in 14 out of 16 *M. haemolytica *strains belonging to MLEE lineage A (Table 3). In the two other strains belonging to MLEE lineage A as well as strains belonging to MLEE lineages B (*n *= 7) and C (*n *= 5), there were only two repeat units per promoter (Table 3).

**Table 3:** Distribution of repeat tract polymorphisms in clonally distinct *M. haemolytica *lineages

Strain ID	Serotype	Host	Country	MLEE lineage^a^	MLEE type^a^	Number of repeat units per promoter	GenBank accession no.
PH292	A2	*Ovis aries*	UK	C	22	2	[GenBank:EU089994]
PH278	A2	*Ovis aries*	UK	C	21	2	[GenBank:EU089992]
PH202	A2	*Bos taurus*	UK	C	21	2	[GenBank:EU089989]
PH598	A2	*Ovis aries*	UK	C	20	2	[GenBank:EU090008]
PH526	A2	*Ovis aries*	UK	C	19	2	[GenBank:EU090004]
PH196	A2	*Bos taurus*	UK	B	18	2	[GenBank:EU089988]
PH550	A2	*Bos taurus*	Germany	B	17	2	[GenBank:EU090006]
PH494	A2	*Ovis aries*	UK	B	16	2	[GenBank:EU090003]
PH588	A13	*Ovis aries*	UK	B	15	2	[GenBank:EU090007]
PH484	A7	*Ovis aries*	UK	B	14	2	[GenBank:EU090002]
PH396	A7	*Ovis aries*	UK	B	13	2	[GenBank:EU090000]
PH296	A7	*Ovis aries*	UK	B	12	2	[GenBank:EU089995]
PH706	A16	*Ovis aries*	UK	A	11	2	[GenBank:EU090009]
PH66	A14	*Ovis aries*	Ethiopia	A	10	2	[GenBank:EU089987]
PH232	A6	*Ovis aries*	UK	A	9	3	[GenBank:EU089990]
PH284	A6	*Ovis aries*	UK	A	8	4	[GenBank:EU089993]
PH398	A1	*Ovis aries*	UK	A	7	4	[GenBank:EU090001]
PH8	A1	*Ovis aries*	UK	A	6	5	[GenBank:EU089983]
PH238	A9	*Ovis aries*	UK	A	5	4	[GenBank:EU089991]
PH56	A8	*Ovis aries*	UK	A	5	3	[GenBank:EU089986]
PH50	A5	*Ovis aries*	UK	A	5	4	[GenBank:EU089985]
PH388	A7	*Ovis aries*	UK	A	4	4	[GenBank:EU089999]
PH338	A9	*Ovis aries*	UK	A	3	3	[GenBank:EU089996]
PH540	A1	*Bos taurus*	Germany	A	2	4	[GenBank:EU090005]
PH346	A12	*Ovis aries*	UK	A	1	3	[GenBank:EU089997]
PH376	A6	*Bos taurus*	UK	A	1	3	[GenBank:EU089998]
PH30	A1	*Bos taurus*	UK	A	1	4	[GenBank:EU089984]
PH2	A1	*Bos taurus*	UK	A	1	3	[GenBank:EU089982]

^a ^Multilocus enzyme electrophoresis (MLEE) lineages and types have been published previously [2].

**Figure 3:**
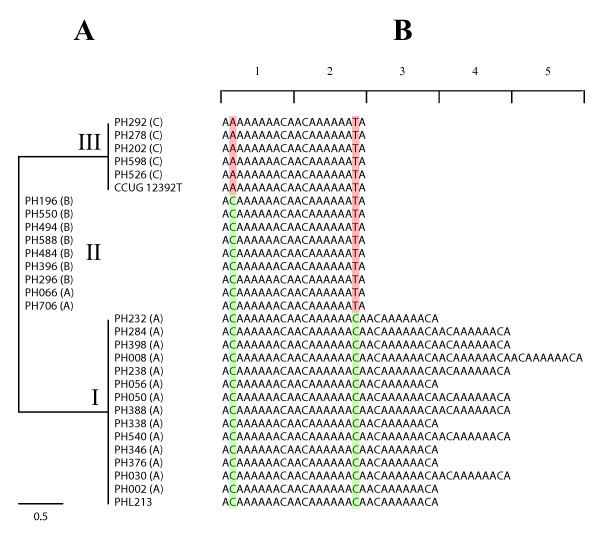
**Evolution of the repeat sequences in *M. haemolytica***. Maximum-parsimony tree of the two distal repeat units from each promoter (A) and repeat tract polymorphisms (B) using *M. haemolytica *strains, which represent the diversity within this species based on MLEE, geographic origin, and host association. See the text for clusters I, II, and III. The green and red boxes highlight polymorphic sites that match and differ, respectively, from the consensus sequence. The number of repeat units per promoter is indicated. Sequence names contain the strain ID and abbreviations of the corresponding MLEE lineage (A, B, or C).

The phylogenetic tree of the distal two repeat units from *M. haemolytica *showed a strong association between the sequence of the repeat units, the number of repeat units per promoter, and the phylogenetic history of this species (Figure 3). First, repeat tracts containing three or more repeat units were restricted to MLEE lineage A as described above and belonged to a cluster (I) of perfect repeat units. Second, repeat tracts containing two repeat units were localised in two clusters (II and III) according to the number of repeat units that varied from the consensus sequence (one and two, respectively) and to the MLEE lineage (B and C, respectively) (Figure 3). These observations are consistent with the generally accepted view that tandem arrays of perfect repeats, such as those present in *M. haemolytica *strains belonging to MLEE lineage A, are hotspots for replication errors, resulting in high rates of expansions/contractions.

### Role of repeat sequences

*M. haemolytica*, like other usually commensal bacteria, must overcome a variety of environmental hurdles to successfully colonise a host, evade the constitutive and inducible defences of this host, migrate from one within-host habitat to another, and be transmitted to another genetically and immunologically distinct host. Presumably to meet these challenges, some bacteria have highly mutable contingency genes that undergo phenotypic switching (phase shifting) [35].

Although the LktA protein plays a role in evading host immunity in the lungs, it may also function as a nasopharyngeal colonization and survival factor. We propose that, in response to the selective pressures associated with the host immune system, expression of the LktA protein is maintained at a baseline level during nasopharyngeal colonisation within individual hosts and asymptomatic nose-to-nose transmission between hosts, while expression is above the baseline level during pulmonary infection.

We further propose that expansions/contractions in the number of perfect repeat units in the *lkt *promoter are associated with phase-variable expression of the LktA protein. The repeat units contain the characteristic sequences of the so-called A-tracts, which are in phase with the helical repeat (i.e. at 10–11 bp intervals). These upstream A-tracts function together as transcriptional enhancers and appear to exhibit inherent DNA curvature [22,23]. Although the precise mechanism of action of these bacterial enhancers is still unknown, this theory is at least partly supported by previous empirical works [36-38], which demonstrated a strong, but not necessarily direct, relationship between the number of A-tracts per promoter and the level of promoter strength.

The hypothesis that phase shifting is correlated with pulmonary infection rests on the idea that virulence is a consequence of the within-host evolution for proliferation into new tissues. We tested this hypothesis retrospectively by analysing the frequency distribution of genetic variants among 17 *M. haemolytica *serotype A1 strains isolated from the two within-host habitats (Table 4).

**Table 4:** Distribution of repeat tract polymorphisms using *M. haemolytica *serotype A1 strains collected from the nasopharynx of healthy cattle and the lungs of cattle with pulmonary infection

Strain ID	Country	Farm	Pulmonary infection	Habitat	OMP^a^	LPS^a^	Number of repeat units per promoter	GenBank accession no.
PH162	UK	1	Yes	Lungs	1.1	1	3	[GenBank: FJ411257]
PH164	UK	2	Yes	Lungs	1.2	1	3	[GenBank: FJ411258]
PH166	UK	2	Yes	Lungs	1.2	1	3	[GenBank: FJ411259]
PH168	UK	3	Yes	Lungs	1.3	1	3	[GenBank: FJ411260]
PH172	UK	4	Yes	Lungs	1.2	1	4	[GenBank: FJ411261]
PH174	UK	4	Yes	Lungs	1.2	1	4	[GenBank: FJ411262]
PH220	UK	?	Yes	Lungs	1.2	1	3	[GenBank: FJ411263]
PH176	UK	5	No	Nasopharynx	1.4	1	3	[GenBank: FJ411264]
PH178	UK	5	No	Nasopharynx	1.4	1	3	[GenBank: FJ411265]
PH180	UK	5	No	Nasopharynx	1.2	1	3	[GenBank: FJ411266]
PH182	UK	5	No	Nasopharynx	1.4	1	3	[GenBank: FJ411267]
PH184	UK	5	No	Nasopharynx	1.2	1	3	[GenBank: FJ411268]
PH186	UK	5	No	Nasopharynx	1.4	1	3	[GenBank: FJ411269]
PH188	UK	5	No	Nasopharynx	1.4	1	3	[GenBank: FJ411270]
PH190	UK	5	No	Nasopharynx	1.4	1	3	[GenBank: FJ411271]
PH192	UK	5	No	Nasopharynx	1.4	1	3	[GenBank: FJ411272]
PH194	UK	5	No	Nasopharynx	1.4	1	3	[GenBank: FJ411273]

^a ^Outer membrane protein (OMP) and lipopolysaccharide (LPS) types have been published previously [53].

The alignment showed significant sequence and positional conservation over the entire 406-bp stretch among all strains, with the exception that they contained different numbers of repeat units per promoter (data not shown). All strains isolated from the nasopharynx of healthy cattle and representing at least two epidemiological clones based on outer membrane protein (OMP) typing (OMP types 1.2 and 1.4) contained three perfect repeat units per promoter (Table 4). The majority of strains isolated from the lungs of cattle with pulmonary infection and representing at least three epidemiological clones (OMP types 1.1, 1.2, and 1.3) also contained three perfect repeat units per promoter, with the exception of two strains (both OMP type 1.2), which contained four perfect repeat units per promoter (Table 4).

The presence of three repeat units per promoter in strains from both within-host habitats suggests that this pattern is associated with a commensal lifestyle in the nasopharynx and that the occasional movement of these bacteria into the lungs may be due to coincidental spill over from the nasopharynx. By contrast, strains containing four repeat units per promoter were confined to the lungs. This observation supports the view that genetic change(s) is (are) required for successful colonisation of the lungs under certain conditions.

Consider an inoculum of *M. haemolytica *that has entered the nasopharynx of a host. Furthermore, assume that successful colonisation of the lungs requires (at least) one change (i.e. addition of one repeat unit) to generate the necessary phenotype (i.e. high-level expression of the LktA protein). Whether or not these mutants exist in the potentially colonising population depends on the total number of cells in the nasopharynx, the fraction of the inoculum that enters the lungs, the rates at which the required genetic change is generated by mutation, and the duration of the carrier state (i.e. the number of generations that survive in the nasopharynx before the population becomes extinct).

This analysis has several potential limitations. First, strain pairs collected from the nasopharynx and lungs of individual hosts with pulmonary infection (rather than from healthy and sick hosts, respectively) should be used to infer within-host evolution. Second, we were unable to perform *in vivo *tests of the predictions generated from this analysis because of the lack of a good animal model for *M. haemolytica*.

### Selection for phase shifting

Whether or not virulence associated with phase shifting will be selected at an epidemiological level depends on the trade-off between the transmission advantage accruing to the cell during short bouts of symptoms (sneezing and coughing) and the loss in long-term asymptomatic nose-to-nose transmission due to either increased clearance, rapid host death, or reduced fitness in the nasopharynx, which is the port-of-entry in new hosts.

If symptoms (sneezing and coughing) and transmission are coupled so the more rapid phase shifting (more virulent) bacteria are transmitted at higher rates than the more slow (benign) ones, phase shifting (virulence) would be favoured in the bacterial population. This epidemiological selection model is supported by the apparent evolutionary success of the high virulence *M. haemolytica *serotype A1/A6 [2].

However, if the genetic and phenotypic changes associated with enhanced fitness in the lungs are detrimental to the bacteria back in the nasopharynx, they are unlikely to confer any long-term advantage in their ability to spread from the lungs of one host to the nasopharynx of another unless revertant changes occur. In this case, disease is a dead end for these bacteria and is a consequence of short-sighted within-host evolution.

If the asymptomatic and symptomatic sites overlap as in the case of respiratory disease, sneezing and coughing generated by the lung-adapted phenotype could favour transmission of freeriders that remain adapted to the nasopharynx, even when disease is a dead end (i.e. revertant changes do not occur, thus preventing transmission of revertant cells to the nasopharynx of new hosts). In other words, freeriders benefit from the collective action without paying the necessary cost associated with the transmission-enhancing feature.

Finally, we present a second model of the epidemiology and within-host infection dynamics of *M. haemolytica *not considered above. Our association analysis showed that the nucleotide change leading to the origin of short tandem arrays of perfect repeat units coincided with the evolutionary success of *M. haemolytica *strains belonging to MLEE lineage A, including relatively benign ones that colonise the ovine nasopharynx asymptomatically and rarely cause disease. To understand the selective forces responsible for phase shifting in these bacteria, which are not invariably pathogenic, it is important to consider its relevance to the commensal existence of these bacteria at an epidemiological level, as a mechanism to facilitate colonisation of genetically and immunologically diverse hosts.

## Conclusion

The continuous circulation of *Mannheimia *bacteria in host populations seems to depend on their capacity for asymptomatic nose-to-nose transmission, although other routes of transmission may exist for some species. Our analysis suggests that the origin of short tandem arrays of perfect repeat units have contributed to the derived virulence phenotype of *M. haemolytica *serotype A1/A6 by allowing phase-variable expression of the LktA protein. Further studies are required to better understand the epidemiology of these opportunistic pathogens. Areas of study should include testing the predictions generated from this analysis using both prospective (experimental) and retrospective (epidemiological) methods.

## Methods

### Taxa used

For this study we included *M. haemolytica *serotype A1 str. PHL213, *M. haemolytica *serotype A2 str. CCUG 12392^T^, *M. glucosida *serotype A11 str. P925^T^, *M. ruminalis *str. HPA113, *M. granulomatis *str. P1135/26^T^, and *M. varigena *str. 177^T ^to balance the number of representatives from the five species within genus *Mannheimia *(Table 1). These strains have been represented in previous studies by Angen et al. [1,39-41] and Larsen et al. [20,21].

### Sequence analysis

The intergenic regions upstream of the *lkt *genes were aligned using Dialign 2 [42] with default settings. This segment-based multiple alignment program is known to produce better alignments for the purpose of phylogenetic footprinting than does the tree-based global multiple alignment program ClustalW [43], because it starts by identifying short conserved regions and then incorporates them into a multiple alignment [44].

### Estimation of evolutionary rates for CP sequences

The relationships of the strains in this study were inferred with 16S rRNA; these sequences have been used successfully for systematic studies in genus *Mannheimia *[1,20]. The 16S rRNA sequences were aligned using Dialign 2 [42] with default settings.

Phylogenetic inference from 16S rRNA and CP sequences (Figure 2) was done under the criterion of maximum-likelihood (ML) using PAUP* version 4.0b10 for UNIX [45]. The best fitting model for both data sets was Hasegawa, Kishino, and Yano's [46,47] model with a discrete four-category gamma distribution [47] of substitution rate among different nucleotide sites (HKY85 + Γ) based on the Akaike Information Criterion (AIC) computed by the programs Modeltest version 3.06 [48] and PAUP* version 4.0b10 for UNIX [45].

We estimated all HKY85 + Γ model parameters (nucleotide frequencies, substitution rates (*μ*), branch lengths (*ν*), gamma shape parameter) from the 16S rRNA data set. The 16S rRNA tree topology was also found to be the single best estimate when using the program MrBayes version 3.0B4 [49], having a posterior probability (PP) of 100%. The tree was rooted using the *M. varigena *str. 177^T ^sequence as outgroup.

We then used the 16S rRNA tree topology as the basis for determining the ML estimates of the HKY85 + Γ model parameters from the CP data set, assuming that the 16S rRNA tree topology is a correct representation of the phylogeny connecting the investigated strains. The evolutionary rates for CP sequences were tested for deviation from expectations derived from a neutral evolution model. The null hypothesis is that the 16S rRNA and CP sequences have evolved under neutral evolution on the same tree topology but at different rates. If this is the case, we would expect the CP tree to be a scaled version of the 16S rRNA tree (with some noise due to the stochasticity in the substitution process). The null hypothesis predicts the following relationship between the expected length of any given branch on the CP tree and the observed length of the corresponding branch on the 16S rRNA tree:



where *T *is the total tree length.

To test whether the observed branch lengths on the CP tree deviate significantly from what we expect under the null hypothesis of neutral evolution, we performed a so-called parametric bootstrapping analysis [50,51]. The program evolver from the PAML package [52] was used to construct 1,000 synthetic "CP-like" data sets by simulating sequence evolution on the CP tree and using the HKY85 + Γ model parameters estimated from the real CP data set. These synthetic "CP-like" data sets are representatives of CP sequences that have evolved under neutral evolution and they can therefore be used to test whether the observed length of any given branch on the CP tree deviates significantly from what we would expect under the null hypothesis of neutral evolution. We used the program BASEML from the PAML package [52] to perform a ML analysis for each of the 1,000 synthetic "CP-like" data sets using the HKY85 + Γ model. For each branch in the 1,000 synthetic "CP-like" trees, we then computed the difference between the observed and the expected branch lengths and tested whether these differences were significantly different from zero (the value expected under the null hypothesis of neutral evolution) by calculating two-tailed *p*-values adjusted by the Bonferroni correction for multiple tests (as we have no *a priori *expectation whether the variation of the difference between the observed and the expected branch lengths should be greater (diversifying evolution) or smaller (unifying evolution) than zero, this test is two-sided).

### Phylogenetic analysis of repeat sequences in *M. haemolytica*

The number of repeat units per promoter was examined using 28 *M. haemolytica *strains representing the diversity within this species based on MLEE, geographic origin, and host association (Table 3) and 17 *M. haemolytica *serotype A1 strains collected from the nasopharynx of healthy cattle and the lungs from cattle with pulmonary infection (Table 4). These strains have been represented in previous studies by Davies et al. [2] and McCluskey et al. [53], respectively. To amplify the intergenic regions in these strains, we designed the forward primer lktpro_UP (5'-CCACACACCCGAATAAAAGGGTCAAAAGTG-3') and the reverse primer lktpro_DOWN (5'-GGAGTTCATCCATAGCCAAGTAATGTTTCC-3') from conserved sequences in the 5' and 3' flanking genes between the *M. haemolytica *+ *M. glucosida *strains. The reaction conditions were 2.5 U *Taq *polymerase, 16 mM (NH_4_)_2_SO_4_, 67 mM Tris-HCl, 0.01% Tween-20, 2.5 mM Mg_2_SO_4_, each primer at 0.5 mM, and each nucleotide at 0.1 mM. The cycling conditions were initial denaturation at 94°C followed by 25 cycles of 94°C for 30 s, 52°C for 30 s, and 72°C for 30 s, finishing with extension at 72°C for 10 min. These PCR products were then directly sequenced.

A phylogenetic tree of the two distal repeat units from 30 *M. haemolytica *strains (Tables 1 and 3) was reconstructed using the maximum-parsimony method as implemented in the program PAUP* version 4.0b10 for UNIX [45]. The first site of the unit sequence was defined arbitrarily by using the *Alu*I restriction site as a landmark [23].

## Authors' contributions

JL carried out the molecular genetic studies, participated in the sequence annotations and in the interpretation of the results, and drafted the manuscript. AGP conducted the phylogenetic analysis of repeat sequences and estimated the evolutionary rates for CP sequences. RLD was responsible for the additional *M. haemolytica *strain collections and participated in the interpretation of the results. PK and JF participated in the design of the study. HC participated in the analysis of gene order data. MB was responsible for the primary strain collection and participated in the interpretation of the results. JEO conceived of the study, participated in its design and coordination, and helped to draft the manuscript. All authors read and approved the final manuscript.
